# Education Research: Educational Informatics: Characterizing Child Neurology Resident Clinical Experiences Using Electronic Health Record Data

**DOI:** 10.1212/NE9.0000000000200334

**Published:** 2026-06-26

**Authors:** Rachel Gottlieb-Smith, Joshua Adams, Miya E. Bernson-Leung, Brian Stamm, Mark V. Mai, Robert Thompson Stone, Elizabeth T. Troy, Kathryn Xixis, Bittu Majmudar-Sheth, Louis T. Dang, Adam C. Dziorny

**Affiliations:** 1 Department of Pediatrics, University of Michigan, Ann Arbor;; 2 Department of Neurology, Boston Children's Hospital, Harvard Medical School, MA;; 3 Department of Neurology, University of Michigan, Ann Arbor;; 4 Department of Pediatrics, Emory University School of Medicine, Atlanta, GA;; 5 Department of Neurology, Golisano Children's Hospital, University of Rochester, NY;; 6 Section of Child Neurology, Department of Pediatrics, Children's Hospital of Colorado, University of Colorado, Denver;; 7 Department of Neurology, University of Virginia School of Medicine, Charlottesville;; 8 Department of Pediatrics, Indiana University School of Medicine, Indianapolis; and; 9 Department of Pediatrics, Golisano Children's Hospital, University of Rochester, NY.

## Abstract

**Background and Objectives:**

Gaps in preparedness for independent practice are common among recent graduates. Clinical experiences serve as the foundation for learning during residency, yet the breadth and variability of clinical exposure of child neurology residents remain poorly characterized. To inform programmatic curricular development and precision medical education (PME), we must better characterize learners' clinical exposure during training. We aimed to use electronic health record (EHR) data to catalog resident clinical experiences, linking diagnostic codes to educational benchmarks.

**Methods:**

We performed a retrospective cohort study of 11 child neurology residents at a single institution in their neurology years of training between July 2024 and May 2025. We used an established algorithm to determine which patient encounters were most meaningful for resident education, based on EHR interaction, and extracted International Classification of Disease (ICD)-10 codes from these encounters. We mapped ICD-10 categories to American Board of Psychiatry and Neurology (ABPN) child neurology certification examination content areas. We identified the most frequent ICD-10 categories seen in inpatient and outpatient settings. We also identified the frequency of ABPN content areas seen across encounters and compared with their relative frequency on the certification examination.

**Results:**

We identified 3,109 meaningful outpatient and 4,469 meaningful inpatient encounters over an 11-month period. The crosswalk between ICD-10 categories and ABPN content areas contains 385 ICD-10 categories mapped to ABPN content domains. There was 90.0% ICD-10 category capture for outpatient encounters and 73.8% for inpatient encounters. The most frequent ICD-10 categories were “epilepsy and recurrent seizures” and “migraine” (outpatient) and “cerebral infarction” and “convulsions” (inpatient). More than half of ABPN content areas were underrepresented in clinical exposure compared with the certification examination. There was overrepresentation of 3 ABPN content areas: “epilepsy and episodic disorders,” “vascular neurology,” and “headache and pain disorders.”

**Discussion:**

A novel crosswalk between ICD-10 categories and ABPN content areas for child neurology enables transformation of EHR-coded data to educationally meaningful information. Using this crosswalk to characterize resident experiences from EHR data may inform rational curricular design and PME. Programs may use these data to optimize rotations and target experiential learning gaps at individual and program levels, resulting in better preparation for autonomous practice.

## Introduction

Residency programs must ensure trainees are ready for autonomous clinical practice. However, gaps in preparedness among recent graduates are common, particularly in patient care competencies,^1^ due to both individual variation in experience and overall curriculum gaps. Inspired by precision medicine, the concept of precision medical education (PME) seeks to address these gaps by identifying needs and delivering customized learning experiences in rapid cycles enabled by data and analytics.^2^ To implement PME, we must better understand the experiential learning that occurs through residents' clinical interactions with patients.^3-8^ These clinical interactions comprise the concrete experiences that serve as the foundation for learning under Kolb's experiential learning theory.^9^

The data to characterize meaningful clinical interactions in near real-time exist in the electronic health record (EHR),^3^ yet these data have not been routinely extracted, cataloged, or interpreted to optimize child neurology resident education. Without an automated system, and with the impracticality of manual case logs on a scale of thousands of patients, we do not have access to these important educational metrics to identify gaps; we instead rely on incomplete and delayed measures, including milestone assessments, in-training examinations, and certification examinations. By the time a graduate fails a certification examination, they are no longer in residency to receive targeted education.

Previous work among pediatric residents has established the framework to identify meaningful clinical encounters for individual residents in inpatient and outpatient settings.^3^ Mai et al.^3^ developed an algorithm called Trainee Individualized Learning System (TRAILS) that uses data elements from each EHR interaction—including time-in-chart, note authorship, order placement, care team membership, and chart closure—to predict which resident-patient encounters are clinically meaningful experiences for pediatric residents. For example, spending more time in the chart, writing a note, and placing an order predict an interaction to be clinically meaningful across all settings. Other predictors include closing the chart (outpatient setting) and care team assignment (inpatient setting). Although the model was developed with validity evidence in pediatric residents only, the algorithm could be used by and potentially adapted for other specialties, including child neurology, to predict meaningful educational experiences for individual residents.

Once meaningful encounters are identified, diagnostic codes from those encounters can be extracted and cataloged to describe each learner's experiential profile. However, diagnostic codes by themselves are insufficient as an educationally meaningful characterization for trainees and training programs. Diagnostic codes need to be translated to a more educationally meaningful language. Previous work in internal medicine serves as a model for translation: a recently developed a crosswalk translates Internal Classification of Disease (ICD)-10 codes to American Board of Internal Medicine content areas (similar to a bilingual dictionary).^10^ No publicly available similar broad educational taxonomy exists for child neurology.

In our multisite collaboration called Precision Learning Across Neurology–Resident Education (PLAN-R), our vision is to develop a timely educational dashboard of child neurology residents' clinical experiences based on EHR data, allowing programs to promptly tailor their residents' clinical experiences to target gaps. To develop the dashboard, we will build on previous work in other specialties, combining (1) identification of meaningful clinical encounters through EHR data elements and (2) mapping diagnostic codes extracted from those encounters to educationally meaningful units for child neurologists.

The primary objective of this study was to develop and validate a child neurology-specific crosswalk, mapping ICD-10 codes to American Board of Psychiatry and Neurology (ABPN) dimension 1 (“Neurologic Disorders and Topics”) content specifications for the certification examination for Neurology With Special Qualification in Child Neurology.^11,12^ The crosswalk will then be piloted at a single institution to characterize child neurology resident experiences.

## Methods

### Trainee Cohort and Data Extraction

We performed a retrospective cohort study of 11 child neurology residents at the University of Michigan who were in their neurology years of training (4 postgraduate year 3 [PGY4], and 4 PGY5 residents) between July 2024 and May 2025. Residents all complete a shared set of core rotations across 3 years, in accordance with Accreditation Council for Graduate Medical Education (ACGME) and program-specific guidelines, and choose individual inpatient, outpatient, and nonclinical electives. Residents were engaged in patient care across child and adult neurology inpatient and outpatient contexts at a single health care system. The same EHR was used across all rotations. We extracted EHR data elements related to clinical work, including time-in-chart, note authorship, order placement, care team membership, chart closure, and ICD-10 diagnostic codes for all patient encounters. Using TRAILS, the previously validated software system with established weights and model thresholds for pediatric residents,^3,4^ we identified meaningful clinical interactions for individual child neurology residents based on features of each discrete resident-patient electronic interaction.

To create content mappings, we extracted encounter diagnoses associated with each interaction. For outpatient (“office visit”) encounters, we identified the primary and secondary encounter diagnoses. For inpatient hospitalization encounters, initially we identified the billing diagnoses from the hospitalization. After initial mapping included a high proportion of nonneurologic diagnoses, we limited our diagnoses to those primary diagnoses billed by neurology attending providers during the hospitalization. We mapped all EHR-specific diagnoses to ICD-10 codes using codes specified within our EHR (Epic Systems, Verona, WI).^13^ See eMethods for additional data extraction details.

### Mapping ABPN Dimension 1 Content Specifications to ICD-10 Categories

Three authors (R.G.-S., J.A., and B.S.), 2 child neurologists and 1 adult neurologist, expanded terminology from the 2023 ABPN dimension 1 child neurology content specifications to enable robust mapping.^11^ For example, in the ABPN content outline, under “movement disorders,” the subcategory of “tremor” is further delineated to 3 subcategories: “essential tremor,” “physiological tremor,” and “drug-induced tremor.” To support capture of additional ICD-10 diagnoses related to tremor, we included additional specific tremor types in the expanded terminology (eTable 1). We attempted to automate and refine the terminology expansion process using an artificial intelligence platform, but the results were not comprehensive nor reliable when compared with manual expansion; we instead completed the terminology expansion manually. Another author (A.D.), a clinical informatics expert, then used Python (version 3) to text match the expanded terminology to the full ICD-10-CM (Clinical Modification) alphabetic index of terms. Terms were then collapsed to ICD-10-CM categories (the unique first 3 alphanumeric digits of the ICD-10-CM codes), identifying 1,038 potential ICD-10-CM category-ABPN terminology pairs.

Potential matches were reviewed by 3 child neurologists (R.G.-S., K.X., and B.M.S.) for concordance, with disagreements reconciled by consensus discussion. During the matching process, R.G.-S., K.X., and B.M.S. noted that not all of the ABPN dimension 1 content specifications initially had complete mapping to all expected ICD-10 categories. The 3 authors then reviewed ABPN content areas from the 2024 Blueprint^12^ and manually identified any missing ICD-10 categories. Any proposed new matches were reviewed, and disagreements again reconciled by consensus discussion.

### Crosswalk Validation

Our initial goal was to capture 95% of ICD-10 categories for resident encounters in our crosswalk (meaning that 95% of ICD-10 categories extracted from resident encounters would be translated to ABPN content areas through the crosswalk table), in line with the established internal medicine crosswalk.^10^ In our first review of ICD-10 outpatient encounter diagnoses among our cohort between July 2024 and January 2025, fewer than 70% of outpatient encounter diagnoses were captured by the crosswalk. R.G.-S., K.X., and B.M.S. reviewed all unmatched diagnoses and added 5 ICD-10 categories to the crosswalk through consensus discussion. In reviewing all unmatched ICD-10 diagnoses, we recognized that our target capture threshold needed to be lowered from 95%, given that many ICD-10 diagnoses were (1) not primarily neurologic (e.g., “immunodeficiency”) or (2) not specific enough to map to a single ABPN content area (e.g., “hemiplegia”). We set a new target capture of ≥90% of primary outpatient encounter diagnoses after intentionally excluding these codes. We then reviewed primary ICD-10 outpatient encounter diagnoses seen by PGY3-5 child neurology residents between July 2024 and April 2025. One additional ICD-10 category was added from unmatched codes. We also reviewed inpatient ICD-10 encounter diagnoses for PGY3-5 child neurology residents between July 2024 and April 2025, as billed by supervising adult or child neurology attendings. Given the higher percentage of nonspecific or nonprimary neurologic diagnoses (e.g., “other sepsis” and “shock—not elsewhere classified”) in the inpatient setting, we decided to set the target capture of ≥70% for inpatient billing diagnoses. We reviewed all unmatched inpatient codes from July 2024 to May 2025 and added 8 ICD-10 categories to the crosswalk. Using our final updated crosswalk, we identified the most frequent ABPN content areas mapped from meaningful clinical interactions for our cohort. We compared ABPN content areas frequencies with the expected frequencies based on the ABPN certification examination content blueprint. We considered percentage differences of ≥1.5% to reflect meaningful differences in clinical vs examination representation; we chose 1.5% because this was the average percentage of the lowest represented content area (autonomic nervous system disorders) on the examination.

### Standard Protocol Approvals, Registrations, and Patient Consents

This study was approved by the University of Michigan Institutional Review Board with a waiver of informed consent.

### Data Availability

Anonymized data not published within this article will be made available by request from any qualified investigator, in accordance with institutional policy.

## Results

Of 9,919 total outpatient encounters between July 2024 and May 2025 for 11 PGY3-5 child neurology residents, the TRAILS algorithm identified 3,109 encounters that were considered meaningful (Table 1). The count of outpatient encounters per resident ranged from 176 to 400 (median: 282). The median patient age across each resident's outpatient encounters ranged from 9.1 to 15.8 years.

**Table 1:** Patient Demographics—Child Neurology Resident Outpatient Encounters by PGY Class (July 2024–May 2025)

PGY class	Number of residents	Total encounter count	Age (median)	Age (mean)	Age (SD)	% Adolescent	% Adult	% Female	Mean Dx per enc
3	4	1,001	14.2	18.3	18	27	30	52	2.3
4	3	861	11.7	11.2	7	34	14	49	2.4
5	4	1,247	9.3	9.9	6	30	11	53	2.0

Abbreviations: Dx = diagnosis; Enc = encounter; PGY = postgraduate year.

Summarizes outpatient encounter volumes and patient characteristics identified through the trainee-patient matching algorithm for each PGY class. For each PGY class, the total number of encounters, patient age distribution (median, mean, and SD), percentage of adolescent patients (ages 12–17 years), percentage of adult patients (age 18+ years), percentage of female patients, and mean number of coded diagnoses per encounter are shown.

There were 16,361 total inpatient encounters between July 2024 and May 2025 for the same 11 PGY3-5 child neurology residents. The TRAILS algorithm identified 4,469 inpatient encounters that were considered meaningful (Table 2). The range of inpatient encounter numbers per resident was 74 to 871 (median: 248). The median patient age across each resident's inpatient encounters ranged from 2.0 to 64.0 years.

**Table 2:** Patient Demographics–Child Neurology Resident Inpatient Encounters by PGY Class (July 2024–May 2025)

PGY class	Number of residents	Total encounter count	Age (median)	Age (mean)	Age (SD)	% Adolescent	% Adult	% Female	Mean Dx per enc
3	4	3,255	63.1	56.4	24	4	90	52	7.2
4	3	400	4.1	7.1	8	23	6	57	13.7
5	4	814	9.3	9.9	11	31	10	50	11.3

Abbreviations: Dx = diagnosis; Enc = encounter; PGY = postgraduate year.

Summarizes inpatient encounter volumes and patient characteristics identified through the trainee-patient matching algorithm for each PGY class. For each PGY class, the total number of encounters, patient age distribution (median, mean, and SD), percentage of adolescent patients (ages 12–17 years), percentage of adult patients (age 18+ years), percentage of female patients, and mean number of coded diagnoses per encounter are shown.

Our completed crosswalk includes 385 unique ICD-10 categories mapped to ABPN content domains (eAppendix 1). There was 90.0% ICD-10 category capture for the 3,109 outpatient encounters and 73.8% for the 4,469 inpatient encounters.

**Figure 1:**
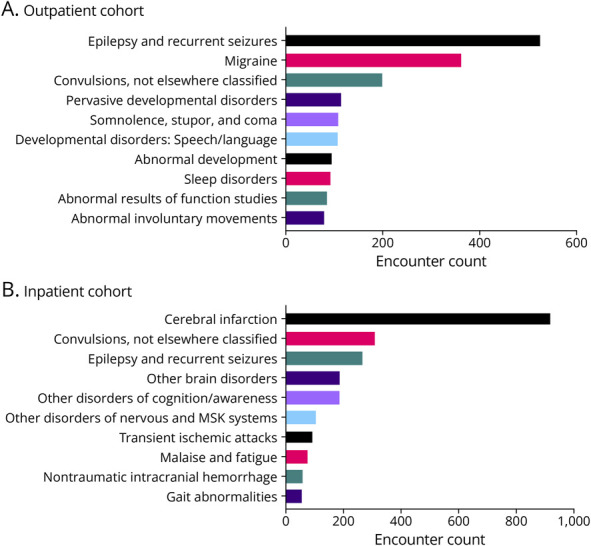
Most Frequent ICD-10 Categories Across Resident Encounters Developmental disorders: speech/language, specific developmental disorders of speech and language; abnormal development, lack of expected normal physiologic development in childhood and adults; other brain disorders, other disorders of brain; other disorders of cognition/awareness, other symptoms and signs involving cognitive functions and awareness; other disorders of nervous and MSK systems, other symptoms and signs involving the nervous and musculoskeletal systems; transient ischemic attacks, transient cerebral ischemic attacks and related syndromes; nontraumatic intracranial hemorrhage, other and unspecified nontraumatic intracranial hemorrhage; gait abnormalities, abnormalities of gait and mobility. The number of encounters (x-axis) for the 10 most frequent ICD-10 categories (y-axis) in the outpatient (panel A) and inpatient (panel B) settings. For the inpatient context, the ICD-10 categories are restricted to those that were coded by a supervising neurologist.

Figure 1 shows the most frequent ICD-10 categories in the inpatient and outpatient settings. In the outpatient setting, the most frequent ICD-10 categories across all patients were “epilepsy and recurrent seizures” (coded in 527 meaningful encounters, 452 as the primary diagnosis) and “migraine” (coded in 362 meaningful encounters, 279 as the primary diagnosis). In the inpatient setting, the most frequent neurology-coded ICD-10 categories across all patients were “cerebral infarction” (922 encounters) and “convulsions, not elsewhere classified” (309 encounters). Most of the “cerebral infarction” encounters (878/922, 95%) were for adult patients. eFigure 1 displays the stratification of the most frequent ICD-10 categories by patient age younger than 18 years and 18 years or older.

**Figure 2:**
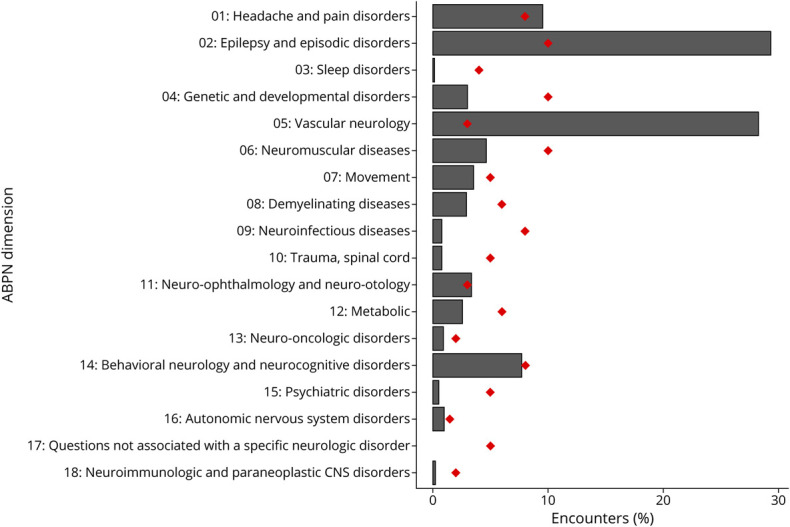
Distribution of American Board of Psychiatry and Neurology Content Areas Across Resident Encounters Neuro-ophtho = neuro-ophthalmology; ABPN = American Board of Psychiatry and Neurology. The percentage of matched American Board of Psychiatry and Neurology (ABPN) dimension 1 (“Neurologic Disorders and Topics”) content specifications for the certification examination for Neurology With Special Qualification in Child Neurology seen across all meaningful resident inpatient and outpatient encounters, after excluding unmatched ICD-10 categories. Red diamonds represent the average percentage of the 400 questions on the certification examination related to that ABPN content area.

Figure 2 shows the distribution of matched ABPN dimension 1 content areas across all meaningful resident inpatient and outpatient encounters, compared with the frequency of the 2025 dimension 1 content areas on the child neurology certification examination.^12^ Overrepresented dimension 1 content areas in clinical exposure compared with the certification examination were “headache and pain disorders,” “epilepsy and episodic disorders,” and “vascular neurology.” “Movement disorders,” “neuro-ophthalmologic and neuro-otologic disorders,” “neuro-oncologic disorders,” “behavioral neurology and neurocognitive disorders,” and “autonomic nervous system disorders” had roughly equal representation (within 1.5% difference). All other content areas were underrepresented in clinical exposure compared with the certification examination.

**Figure 3:**
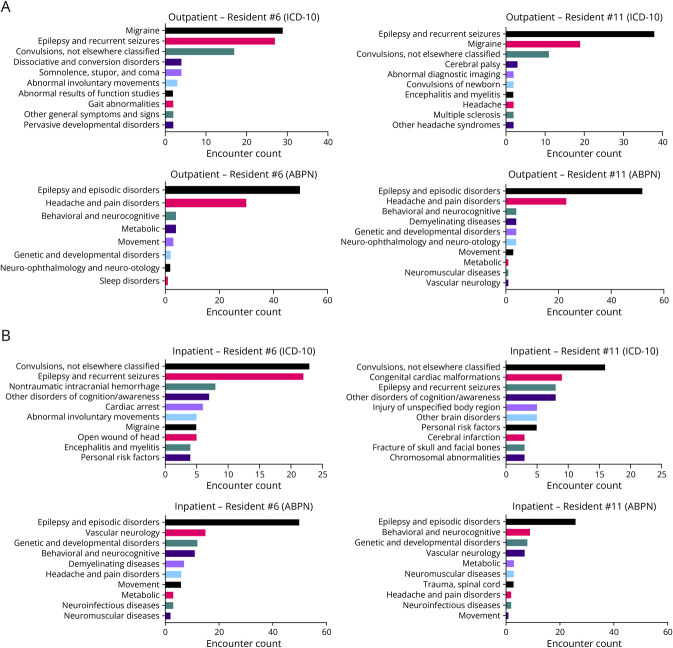
Comparison of Most Frequent ICD-10 Categories and American Board of Psychiatry and Neurology Content Areas Across 2 Residents' Encounters Gait abnormalities, abnormalities of gait and mobility; abnormal diagnostic imaging, abnormal findings on diagnostic imaging of other body structures; encephalitis and myelitis, encephalitis, myelitis, and encephalomyelitis; ABPN, American Board of Psychiatry and Neurology; behavioral and neurocognitive, behavioral neurology and neurocognitive disorders; neuro-ophtho, neuro-ophthalmology; nontraumatic intracranial hemorrhage, other and unspecified nontraumatic intracranial hemorrhage; other disorders of cognition/awareness, other symptoms and signs involving cognitive functions and awareness; personal risk factors, personal risk factors, not elsewhere classified; congenital cardiac malformations, congenital malformations of aortic and mitral valves; other brain disorders, other disorders of brain; chromosomal abnormalities, monosomies and deletions from the autosomes, not elsewhere classified. Comparison of most frequent ICD-10 categories and American Board of Psychiatry and Neurology content areas across 2 residents' encounters A and B shows the number of encounters (x-axis) for the 10 most frequent ICD-10 and ABPN categories (y-axes) in the outpatient (panel A) and inpatient (panel B) settings for 2 different residents in the same year of training (resident #6 and resident #11). For the inpatient context, the ICD-10 categories are restricted to those that were coded by a supervising neurologist.

Figure 3 displays a comparison in ICD-10 category and ABPN content area exposure of 2 residents in the same year of training, who did not have any gaps in training during the 11-month period. Exposure in the outpatient setting was generally similar; both residents had the greatest exposure to headache and pain disorders and epilepsy, although there was some variability in exposure to other diagnoses such as demyelinating diseases. In the inpatient setting, seizures and epilepsy were the most common areas of exposure of both residents. Frequencies of some top inpatient content areas did vary between residents; for example, 1 resident had greater exposure to intracerebral hemorrhage, while the other had greater exposure to congenital cardiac malformations.

## Discussion

As an initial step in our multisite collaboration to develop a scalable educational dashboard of child neurology resident experiences based on EHR data, we developed a novel crosswalk that maps diagnostic codes to ABPN content specifications for the certifying examination intended to assess preparation for independent practice following relevant clinical training. We identified more than 7,500 meaningful encounters for child neurology residents at a single institution over an 11-month period and then used the crosswalk to map these encounters to educationally relevant content areas.

Notably, on a residency program level, we found discrepancies in frequency of clinical exposure to content areas compared with tested frequency based on the ABPN certification examination. Epilepsy and episodic disorders, headache and pain disorders, and vascular neurology were clinically overrepresented, while many ABPN content areas were relatively underrepresented. This finding is not unique to child neurology; a discrepancy between the experiential clinical curriculum and certifying board examination content area was similarly found for an internal medicine residency program.^6,10^

Knowledge of skewed exposures may guide rational curricular design.^4^ Although we are unable to modify the patient concerns and diagnoses seen in different units across our institutions, we may adjust required rotations at the program level, as allowed within existing ACGME^14^ and ABPN requirements.^15^ For example, given the overrepresentation of vascular neurology within the resident cohort, which is related to adult neurology experiences, we will advocate to reduce inpatient adult neurology time on the dedicated stroke service in favor of other adult neurology inpatient opportunities. We will also review our conference curriculum to ensure that all the underrepresented content areas are covered in nonclinical settings as a supplement and develop additional educational content as needed. In addition, broader implementation of such information technology-enabled insights across child neurology programs could help to inform future modifications of ACGME and ABPN training program requirements and/or certification examination content to promote more optimal alignment between clinical training and summative assessments.

This phase of our work also enabled us to identify variability in individual resident clinical interactions. To maintain resident anonymity, we cannot differentiate the individual residents by specific year of training. We suspect that some of the variability in the number of meaningful encounters, as well as the variability in median patient age, was related to individual trainee schedules (e.g., leave time) as well as variability in rotational exposure by year, with more adult neurology rotations during the PGY3 year. Even within the same year of training, our results also show some variability in the frequency of diagnoses to which individual residents were meaningfully exposed, particularly in the inpatient setting. The PLAN-R system in development will ultimately display clinical exposure distributions for each resident, providing timely insights to both residents and program leadership that enable targeted interventions at the individual trainee level. For example, specific elective rotations (e.g., neuroimmunology or neuroinfectious diseases) may be recommended for individual residents before graduation to ensure adequate exposure. If targeted elective opportunities are limited related to faculty expertise at a particular institution, program leadership may recommend specific training modules, conferences, or away rotations.

Limitations of this study provide opportunities for future research. Clinical exposure is not synonymous with competency, and further work is required to establish the correlation between the 2 constructs. This pilot study was conducted at a single institution with a limited number of residents who have defined program-specific rotation requirements and elective choices; additional research is required to determine the generalizability to other programs. We used an existing logistic regression model (TRAILS) to classify meaningful clinical interactions for individual child neurology residents, which had been previously trained and validated on pediatrics residents at a different institution. Although the output has face validity, the generalizability to child neurology residents has not previously been established. The initial model focused on inpatient and outpatient encounters for pediatrics residents but may not adequately capture the nuances of consulting roles and on-call experiences for child neurology residents. The model also does not capture non–face-to-face encounters, which have increased in frequency in recent years, or secure chat messaging through the EHR, which has recently become a common mode of communication and interaction surrounding patient care. However, despite these model limitations, all included encounters and diagnoses had substantial touchpoints with resident trainees in the EHR. In ongoing PLAN-R project efforts, we are updating touchpoints and recalibrating the logistic regression model to predict meaningful educational interactions for current child neurology residents. We also acknowledge that EHR coding data may not accurately reflect the complete clinical or educational experience related to a resident-patient interaction; restricting the coding data to only the primary or billing diagnoses may further that limitation.

This study is an initial step to more accurately characterize child neurology resident clinical experiences to guide PME. We present a shareable crosswalk that can be used to map ICD-10 categories to an educationally meaningful framework to guide future educational interventions. Through our PLAN-R system, we aim to harness EHR data to target learning gaps through evidence-based individualized learning plans and rational curricular design, resulting in graduates who are competent across all areas of child neurology.

## Glossary

ABPN: American Board of Psychiatry and Neurology
ACGME: Accreditation Council for Graduate Medical Education
EHR: electronic health record
PLAN-R: Precision Learning Across Neurology–Resident Education
PME: precision medical education
TRAILS: Trainee Individualized Learning System
